# Comparison of different screening methods for the selection of Ascochyta blight disease on chickpea (*Cicer arietinum* L.) genotypes

**DOI:** 10.3389/fpls.2024.1347884

**Published:** 2024-03-26

**Authors:** Abdulkadir Aydoğan

**Affiliations:** Head of Food Legumes Breeding, Central Research Institute for Field Crops, Yenimahalle, Türkiye

**Keywords:** artificial epidemic, Ascochyta blight, chickpea, selection, field condition, real-time PCR, molecular characterization

## Abstract

Chickpea (*Cicer arietinum* L.) is the second most important edible food grain legume, widely grown all over the world. However, the cultivation and production of chickpea are mainly affected by the Ascochyta blight (AB) disease, which causes losses of up to 100% in areas with high humidity and warm temperature conditions. Various screening methods are used in the selection of chickpea genotypes for resistance to AB disease. These methods are natural field condition (NFC), artificial epidemic field condition (AEC), marker-assisted selection (MAS), and real-time PCR (RT-PCR). The study was conducted with 88 chickpea test genotypes between the 2014 and 2016 growing seasons. The results of the screening were used to sort the genotypes into three categories: susceptible (S), moderately resistant (MR), and resistant (R). Using MAS screening, 13, 21, and 54 chickpea genotypes were identified as S, MR, and R, respectively. For RT-PCR screening, 39 genotypes were S, 31 genotypes were MR, and 18 genotypes were R. In the AEC method for NFC screening, 7, 17, and 64 genotypes were S, MR, and R, while 74 and 6 genotypes were S and MR, and 8 genotypes were R-AB disease. As a result of screening chickpea genotypes for AB disease, it was determined that the most effective method was artificial inoculation (AEC) under field conditions. In the study, Azkan, ICC3996, Tüb-19, and Tüb-82 were determined as resistant within all methods for Pathotype 1.

## 1 Introduction

Chickpea (*Cicer arietinum L*.) is the second most widely cultivated pulse after dry bean, and it is one of the major plant-derived protein sources for the human diet. It is produced in 153 countries in different parts of the world with over 15 million ha^−1^ with a production of approximately 15.9 million tons. The yield of chickpea is 1,058 kg/ha worldwide, with Turkey accounting for more than 475,000 tons of production (FAO, 2021).

Chickpea cultivation in Turkey is carried out using dry farming methods without irrigation (Bayramoğlu and Gündoğmuş, 2010; Erman et al., 2012). The climatic conditions of the chickpea-growing areas are characterized by low annual total rainfall, low winter temperatures, and hot and dry summers, and the majority (65%) of precipitation falls in winter and spring (Küsmenoğlu and Aydin, 1995). In Turkey, chickpea is grown in both the spring and the autumn seasons. It is cultivated in spring at high altitudes, whereas it is cultivated in fall at lower altitudes (Aydoğan et al., 2009).

The yield and profitability have decreased in chickpea production over the years because of the various biotic and abiotic stressors, as well as the distinctive traits of legumes (Slinkard et al., 2000). Ascochyta blight (AB) disease caused by *Ascochyta rabiei* is the main disease limiting chickpea yield (Küsmenoğlu and Meyveci, 1996), which causes a significant economic downturn (Haware et al., 1986) in more than 40 countries including Turkey (Bhardwaj et al., 2010; Sharma and Ghosh, 2016). Planting time is postponed in Turkey to tackle the detrimental effect of Ascochyta chickpea blight. The epidemic can cause product losses of up to 100%, which indicated the global importance of the disease (Trapero-Casas and Jiménez-Díaz, 1986; Nene and Reddy, 1987; Pande et al., 2005; Singh et al., 2007).

The occurrence and severity of AB disease in cultivated chickpea is highly dependent on weather conditions. It mainly has detrimental effects on the vegetative and podding stages of the crop in regions with cool (15˚C to 25˚C) and humid weather conditions (>150 mm precipitation) during the crop growing season. The pathogen causes severe blight epidemics and substantial yield losses, especially in susceptible cultivars and under favorable disease conditions (Singh and Reddy, 1990; Shtienberg, 2010). Owing to the strong genotype × environment (G × E) interaction, the disease status can vary significantly from year to year depending on the presence of the pathogen in the environment (McDonald and Linde, 2002).

Many studies have been conducted to comprehend the inheritance of the resistance of AB (Tekeoglu et al., 2000; Bhardwaj et al., 2010). The vast majority of research indicates that disease resistance has a quantitative feature and depends on many quantitative trait loci (QTL) (Tekeoglu et al., 2002; Anbessa et al., 2009). Cultural, host plant resistance (use of resistant varieties), chemical control, and biological control methods can be used to combat the disease (Foresto et al., 2023). Preferring resistant cultivars is the most effective and most ecologically beneficial technique among the listed methods (Jimenez-Diaz et al., 1993; Chen et al., 2004; Li et al., 2015).

To date, a high level of stable resistance/immunity against AB has not yet been identified in the chickpea gene pool (Gayacharan et al., 2020; Sharma et al., 2023). *A. rabiei* continues to evolve and hence disrupts the host resistance systems (Nene and Reddy, 1987; Chen et al., 2004; Kanouni et al., 2011). Therefore, even if materials that are tolerant or partially resistant to the AB are developed, they will subsequently be diseased and lose their durability. This could be solved by creating new sources with increased host tolerance and resistance. Hence, developing resistant varieties should be the key goal of chickpea breeding studies, particularly in places where the AB is severe and widespread to sustain cultivation and production.

Various techniques are used to screen breeding materials to develop resistant varieties and lines. In addition to field, greenhouse, and controlled climatic conditions (Pande et al., 2011a; Kaur et al., 2014; Varshney et al., 2014), molecular techniques such as real-time PCR (RT-PCR) and marker-assisted selection (MAS) are also utilized in breeding projects to screen genetic material for the presence of the AB (Kumar et al., 2016).

The field condition artificial inoculation test method used to screen breeding material for disease was developed by ICARDA and ICRISAT (Nene et al., 1981; Singh et al., 1984). Within the method, an observation garden is established with a sensitive control that is placed in every two to four rows. Conidia suspension that is obtained from diseased plants collected from the previous year is sprayed on the plants. Two evaluations are made, the first after the sensitive control dies, and the second during the pod-setting period. In the study that was conducted by ICARDA and ICRISAT, 15,300 acceptance and desi materials were tested for AB in field and greenhouse conditions. Twelve kabuli and 3 desi-type chickpea AB were found to be resistant to Pathotypes 1, 2, 3, and 4 (Singh et al., 1992). A total of 112 materials of kabuli and desi type were tested in 51 locations in different countries. In the trial, lines numbered ILC 72, 191, 3,279, and 3,856 were found to be resistant in 8 of 11 districts (Singh et al., 1984).

In natural conditions, no intervention is made on the spread and severity of the disease, except for the presence of sensitive varieties in the trial. Observations to assess the material for the disease are carried out twice under natural circumstances during podding and harvesting. Under natural and artificial field epidemic conditions, there is a strong genotype × environment (G × E) interaction, which causes the disease state to alter significantly from year to year depending on the presence of the pathogen in the environment (McDonald and Linde, 2002). Environmental conditions significantly affect the severity and prevalence of the disease in the natural field condition (NFC) and artificial epidemic field condition (AEC).

MAS is a further technique for genotype-based AB screening of chickpeas. For qualities that are challenging to select, such as disease resistance and abiotic stressors, MAS is particularly beneficial, because it is simpler than phenological screening, is unaffected by environmental influences, is safer, and enables early selection (Yorgancilar et al., 2015). MAS is frequently used to check breeding material for disease because it is simpler and safer than morphological screening. It is also unaffected by environmental influences and enables early selection. SSR, SCAR (Sequence Characterized Amplified Region), ISSR, and RAPD techniques are used in these scans (Ali et al., 2008). Resistant gene-based markers are developed for the selection of several diseases in various plants, for example, SSR marker for leaf rust (*Puccinia recondita* f. sp. *tritici*) in wheat (Suenaga et al., 2003), SA598 SCAR for Gall midge (*Orseolia oryzae*) in paddy (Sardesai et al., 2002), SCAR and CAPS for sugarcane mosaic virus (SCMV) in maize (Dussle et al., 2002), and CAPS (Graner et al., 2000) for leaf rust (*Puccinia hordei*) in barley. Resistant QTLs have also been identified for AB of chickpea. Many markers are used for MAS (Iruela et al., 2006; Imtiaz et al., 2008; Castro et al., 2015; Sudheesh et al., 2021). It is stated that four SCAR markers are used for QTLs (QTL _AR1_ and QTL _AR2_), which are identified as being associated with the resistance of AB disease caused by *A. rabiei* in the Kabuli × Desi RIL population (Iruela et al., 2006). SCAR markers detect local varieties, advanced breeding lines, disease susceptibility, and resistance of cultivar alleles by 90% (Madrid et al., 2013).

Another screening method used for AB is RT-PCR. Phan et al. (2002) developed a PCR-RFLP assay for the detection of the pathogen in infected leaves or seeds of the host (chickpea) using primers targeting the conserved sequences of the internal transcribed spacer (ITS) regions of *A. rabiei*. The technique is a sensitive method for measuring pathogen DNA. With this technique, the severity of the disease can be determined and the disease can be monitored (Gachon et al., 2004; Schena et al., 2004; Pasche et al., 2013). The method is widely used to detect and identify pathogens, their quantity, disease severity in infected seeds, and host plant tissue (Udupa and Weigand, 1997; Rigotti et al., 2002; Jiménez-Fernández et al., 2011; Leiminger et al., 2015).

The article is an output of the multidisciplinary project titled “Development of Germplasm Tolerant to Chickpea Blight (*A. rabiei*) by Combining Classical and Modern Breeding Techniques”.

In the study, NFC, AEC, MAS, and RT-PCR methods were used to evaluate the resistance of project material against AB. The study aims to compare the screening methods of chickpea breeding materials for AB and to determine the most effective method.

## 2 Materials and methods

### 2.1 Material

The project material consists of 84 advanced chickpea lines (genotypes) and four checks [Çağatay, Gökçe, Azkan (kabuli type), and ICC3996 (desi type)]. A total of 84 genotypes were lines that can be candidates for cultivars. The four checks were cultivars that are widely grown in Turkey and have different levels of disease resistance. These were evaluated for their reaction to AB. Among the lines, Çağatay and Gökçe are susceptible, while Azkan (Aydin et al., 2016) and ICC3996 (Zhou et al., 2019) are resistant to AB. The 50% flowering days of the materials of the experiment varied between 76 and 87 days, whereas the 100-g weight of the materials was between 23.6 and 42.7 g.

### 2.2 Methods

Within the scope of the study, genotypes were screened and evaluated using four screening methods to determine AB disease resistance. These methods were NFC, AEC, MAS, and RT-PCR.

#### 2.2.1 NFC

**Figure 1:**
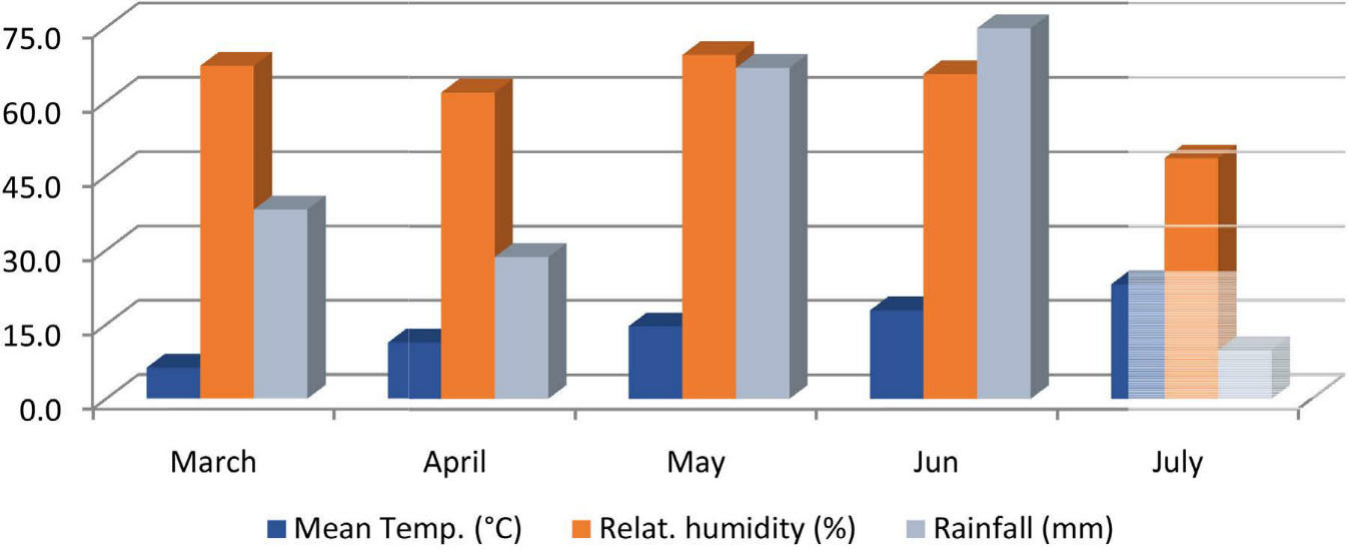
Mean temperatures, relative humidity, and rainfall during 2014 at Haymana, Ankara. Source: TSMS, 2014.

Yield and preliminary yield trials at Haymana, Ankara, Turkey were conducted using three and two replications under field conditions respectively during the 2014 growing season. The plot dimensions are 6 m^2^ (5 m × 0.3 m × 4 rows) and the height of the cultivation area is 1,050 m above sea level. Forty-five seeds were used per square meter. During the cultivation process, the total amount of precipitation was 218.2 mm and the highest precipitation was in June with 74.8 mm. The climate data of the experimental area are given in **Figure 1**. The amount of precipitation was more than the average for many years.

The trials were planted on 6 March 2014. AB observations were taken three times (flowering, podding, and harvest stage). After that, mean AB observations for each line were calculated. Disease scoring was recorded on a 1–9 (1: resistance, 9: susceptible) disease rating scale (Reddy and Singh, 1984). Then, disease scores were modified by Pande et al. (2011a).

#### 2.2.2 AEC

Genotypes of the yield and preliminary yield trials in the breeding program of 2014 were used as material in the experiment with 88 genotypes sown with two replications in 1-m rows under the field conditions as a disease nursery at Haymana, Ankara. The genotypes were sown on 25 March 2014.

The isolates of Pathotype 1 were used as an artificial inoculation source. After 57 days from sowing, the trials were inoculated before flowering time by spraying aqueous spore suspensions having a concentration of 5 × 10^5^ spores/mL. The nursery was inoculated with diseased debris and sprinkler irrigation was provided to create humid conditions (Udupa and Baum, 2003; Chen et al., 2005).

The disease observations were taken in cases where susceptible control genotypes had completely succumbed to AB disease. The evaluation of chickpea genotypes for AB reaction was performed by using a rating scale based on the severity of infection on leaves, stems, and pods as proposed by Reddy and Singh (1984). Disease observations were taken three times during the experiment. Afterwards, the average of three observations was calculated. Then, these scores were grouped as shown in **Table 1**.

**Table 1:** Scoring and classifying for AB disease.

R	1 = No infection
	2 = Highly resistant (1%–5% of plants showed blight)
	3 = Resistant (6%–10% showed blight)
MR	4 = Moderately resistant (11%–15% showed blight)
	5 = Intermediate (16%–40% showed blight)
S	6 = Moderately susceptible (41%–50% showed blight)
	7 = Susceptible (51%–75% showed blight)
	8 = Highly susceptible (76%–100% showed blight)
	9 = All plants died

Source: Reddy and Singh (1984) and Pande et al. (2011a).

#### 2.2.3 MAS

The SCAR Genomic DNA was isolated from the leaves of the 88 genotypes. For DNA isolation, the Gene Matrix Plant Fungi DNA Purification Kit (Cat No. E3595) was used and done according to Kwasniak et al. (2013). DNA quality and quantity measurements were by made using 1% agarose gel and a Nanodrop ND-1000 spectrophotometer. PCR reaction of the three SCAR markers, 15 ng of DNA, 5 pmol forward primer, 5 pmol reverse primer, 0.5 mM total dNTP, 0.5 units of Go Taq DNA Polymerase (Promega) (containing 1.5 mM MgCl_2_), and 3 μL buffer (5× Buffer) were carried out at a total of 15 μL. SCAR-primer sequences are presented in **Table 2**.

**Table 2:** Information on SCAR primers’ sequences.

Marker name	The primer sequences (5′–3′)
SCAE19_336_	Forward: gacagtccctccattatctaaac
SCAE19_336_	Reverse: gacagtccctatgtgtgagaat
SCK13_603_	Forward: ggttgtaccccatcctcccg
SCK13_603_	Reverse: ggttgtacccttgtgccacta
SCY17_590_	Forward: gacgtggtgactatctagc
SCY17_590_	Reverse: gacgtggtgaaaatagatacc

Source: Iruela et al. (2006).

The PCR program used for the PCR reaction (Touchdown) is as follows:

1. Three minutes at 94˚C
2. One minute at 94˚C
3. From 66˚C to 57˚C for 1 min 45 s
4. Two minutes at 4.72˚C
5. It was applied as a total of 21 cycles, 10 min at 5.72˚C.

After PCR, PCR products of loci were visualized on 2% agarose gel and band profiles were determined. The definition of band profiles was made according to Iruela et al. (2006) and Winter et al. (1999). The materials were evaluated at the SCY17_590_ mark, and band profiles showed resistance of genotypes at 590 bp and moderate resistance at the SCY19_336_ mark with 336 bp and susceptibility if there was no band on the SCY19_336_ mark.

In addition, classifying for disease was made in the marker evaluations. In this grouping, they were evaluated as resistant (R) if the genotype was resistant to three markers, as moderately resistant (MR) if it was resistant for one or two markers, and as susceptible (S), if the genotype was susceptible for three markers.

#### 2.2.4 RT-PCR

The pathogen isolate was grown in Petri dishes containing Chickpea-Flour-Dextrose-Agar medium for 14 days in an incubation room at 22˚C ± 1˚C and 12 h of light (near UV) period. Chickpea-Flour-Dextrose-Agar (CSMDA: 40 g of chickpea flour, 20 g of dextrose, 20 g of agar, and 1 L of pure water) medium is the most suitable medium for sporulation.

The concentration of this prepared spore suspension was determined by counting with a thoma slide and dilution with sterile water to 1 × 10^5^ spores/mL. Study materials were grown in pots. Three Petri dishes were used for each inoculation point, and each Petri dish contained 10 leaflets. Detection of *A. rabiei* in plant tissue was made by an RT-PCR method that was reported by Udupa and Weigand (1997) and subsequently developed by Bayraktar et al. (2016). The samples were taken from all genotypes on the 8th day after inoculation. In addition, disease reactions in chickpea leaflets were calculated after each inoculation period. Disease incidence (%) was expressed as the proportion of diseased leaflets. Percent disease severity was evaluated from the affected leaflet size based on a 0–5 scale: 0: no lesion, 1: 10%; 2: 25%; 3: 50%, 4: 75%, and 5: 100% affected leaflets (Dolar et al., 1994). According to the classification, the percentage of disease rate was evaluated into three categories: 0-1: up to 10% as resistant (R), 2–3: 10%–50% as moderately resistant (MR), and 4–5: over 50% as susceptible (S).

In the study, the regression coefficient between the amount of pathogen in the leaf and tissue and the disease severity and incidence was calculated. Principal component analysis (PCA) and Biplot were performed in the JMP statistical program in the methods used in disease testing of genotypes.

## 3 Results

### 3.1 NFC

**Figure 2:**
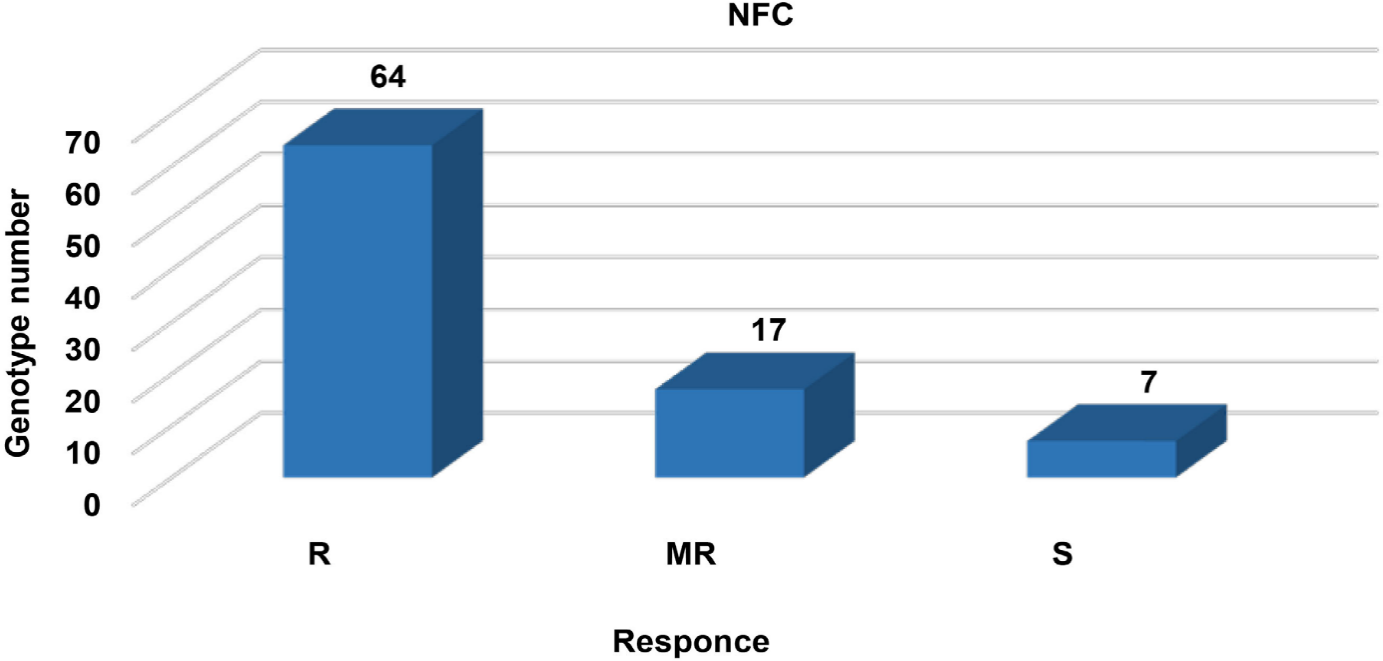
The classification of the disease observation for 88 chickpea genotypes under the natural field condition (NFC).

In the screening method of NFC, approximately 62 (73%), 17 (19%), and 5 (8%) genotypes were classified as R, MR, and S, respectively. While Azkan and ICC 3996 were resistant to AB, Çağatay and Gökçe were found to be susceptible in checks. In this growing season, rainfall, relative humidity, and temperature were not suitable for the occurrence and spread of AB in Haymana. The classifications of the AB disease observation among the genotypes in yield trials of 2014 under the NFC are given in **Figure 2**.

The seasonal rainfall and relative humidity were appropriate for spore production and mycel development but the temperature was not favorable. The stages of flowering and pod filling period were in June in Haymana, which are the most sensitive periods for AB spread. June had good conditions for AB spread with 74.8 mm rainfall and 65.6% relative humidity, in contrast with non-suitable temperatures (17.9˚C). Therefore, the disease did not exist and spread in this season. As a general rule, if rainfall, relative humidity, and temperature are missing or insufficient, the crop is either less affected or not damaged by the AB disease. Hence, NFC had the highest number of resistant materials.

### 3.2 AEC

**Figure 3:**
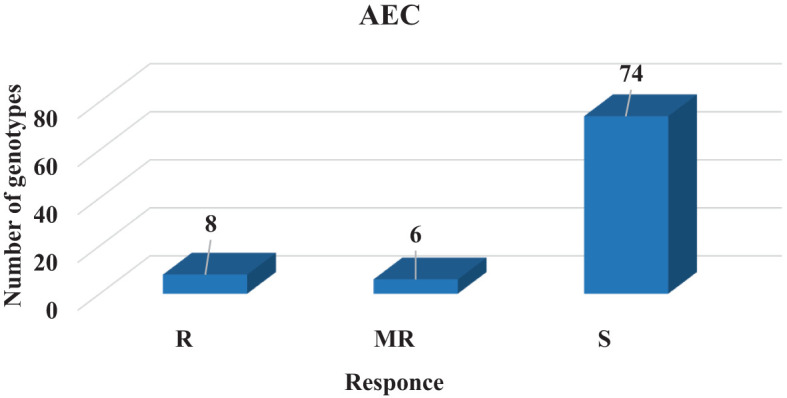
AB observation groups under AEC for screened genotypes.

Artificial inoculation conditions and climatic conditions during the growing season had a positive effect on the development and severity of the disease. A total of 72, 6, and 6 genotypes of a total number of 84 advance lines in AEC were susceptible (scores: 6–9), moderately resistant (scores: 4–5), and resistant (scores: 1–3), respectively (**Figure 3**). Çağatay and Gökçe were identified as susceptible (scores: 8–9) while Azkan and ICC3996 were detected as resistant to the method of AEC. Under AEC, 9% of the genotypes were resistant (R), 7% were moderately resistant (MR), and 84% were susceptible (S). Resistant genotypes of out-of-checks were line Tüb 18, 19, 70, 71, 72, and 82.

### 3.3 MAS

PCR reactions of three SCAR (SCY19_336_, SCK13_603_, and SCY17_590_) markers were studied in 88 chickpea genotypes for MAS against AB in the study, using both agarose gel electrophoresis and capillary electrophoresis conditions. The separation of the genotypes carrying the resistance allele was determined according to the band profiles.

The results showed that 84 genotypes and four standards were scanned with the help of three markers. Approximately 21 sensitive genotypes were classified as moderately resistant (MR), and in 13 genotypes, they were considered susceptible (S) because the resistance alleles could not be determined (S) (**Table 3**). Both markers (SCY19_336_ and SCK13_603_) showed similar efficacy and it was observed that the SCY17_590_ marker determined the genotype with a greater number of resistance alleles. When the markers SCY19_336_, SCK13_603,_ and SCY17_590_ were scanned in agarose gel electrophoresis for comparison among themselves, it was seen that all of these markers showed the presence of resistance alleles, and moderately resistant genotypes were not found.

**Table 3:** The AB resistant and susceptible genotypes using three SCAR markers for the MAS of chickpea germplasm.

Genotype	Molecular screening				Genotype	Molecular screening			
	SCAE19_336_	SCK 13_603_	SCY17_590_	Response*		SCAE19_336_	SCK 13_603_	SCY17_590_	Response*
Tüb-01	+	+	+	R	Tüb-50	+	+	+	R
Tüb-02	+	+	+	R	Tüb-51	+	+	+	R
Tüb-03	+	–	–	MR	Tüb-52	+	–	–	MR
Tüb-04	+	–	–	MR	Tüb-53	+	+	+	R
Tüb-05	+	+	+	R	Tüb-54	–	–	–	S
Tüb-06	+	+	+	R	Tüb-55	–	–	–	S
Tüb-07	+	+	+	R	Tüb-56	+	–	–	MR
Tüb-08	+	+	+	R	Tüb-57	+	–	–	MR
Tüb-09	+	+	–	MR	Tüb-58	+	+	+	R
Tüb-10	+	+	+	R	Tüb-59	+	–	–	MR
Tüb-11	+	+	+	R	Tüb-60	–	–	–	S
Tüb-12	+	+	+	R	Tüb-61	+	+	+	R
Tüb-13	+	+	+	R	Tüb-62	–	–	–	S
Tüb-14	+	+	+	R	Tüb-63	+	+	+	R
Tüb-16	+	+	+	R	Tüb-64	–	–	–	S
Tüb-18	+	+	+	R	Tüb-65	+	+	+	R
Tüb-19	+	+	+	R	Tüb-66	+	+	+	R
Tüb-20	+	+	+	R	Tüb-67	+	+	+	R
Tüb-21	–	–	–	S	Tüb-68	+	+	+	R
Tüb-22	+	+	+	R	Tüb-69	+	+	+	R
Tüb-23	+	–	–	MR	Tüb-70	+	–	–	MR
Tüb-25	+	–	–	MR	Tüb-71	+	–	–	MR
Tüb-26	+	+	–	MR	Tüb-72	+	+	+	R
Tüb-27	+	–	–	MR	Tüb-74	+	+	+	R
Tüb-28	+	+	–	MR	Tüb-75	+	+	+	R
Tüb-29	+	+	–	MR	Tüb-76	+	+	+	R
Tüb-30	+	+	–	MR	Tüb-78	+	+	+	R
Tüb-31	–	–	–	S	Tüb-79	+	+	+	R
Tüb-33	+	+	–	MR	Tüb-82	+	+	+	R
**Çağatay**	+	+	+	R	Tüb-84	–	–	–	S
**Gökçe**	+	+	+	R	Tüb-86	+	+	+	R
Tüb-37	+	–	+	MR	Tüb-87	+	+	+	R
Tüb-38	+	+	+	R	Tüb-93	+	+	+	R
Tüb-39	+	+	+	R	Tüb-96	–	–	–	S
Tüb-40	+	+	+	R	Tüb-97	+	–	+	MR
Tüb-41	+	+	+	R	Tüb-100	+	–	–	MR
Tüb-42	+	+	+	R	Tüb-105	+	+	+	R
Tüb-43	+	+	+	R	Tüb-108	+	+	+	R
Tüb-44	–	–	–	S	Tüb-114	+	+	+	R
Tüb-45	+	+	+	R	Tüb-119	+	+	+	R
Tüb-46	–	–	–	S	Tüb-121	+	+	+	R
Tüb-47	+	+	–	MR	Tüb-124	+	+	+	R
Tüb-48	–	–	–	S	**Azkan**	+	+	+	R
Tüb-49	–	–	–	S	**ICC 3996**	+	+	+	R

Bold values are check (control) genotypes.

In the study conducted with three MAS markers, 61%, 24%, and 15% of the genotypes were evaluated as resistant, moderately resistant, and susceptible, respectively. All checks in this method were identified as resistant genotypes.

### 3.4 RT-PCR

The disease incidence in eight of the chickpea genotypes tested in the study was 100%. Furthermore, 15 genotypes that showed a disease incidence of 0%–10%, 30 genotypes with 11%–40% resistance, and 43 genotypes with 40%–100% were resistant (R), moderately resistant (MR), and susceptible (S) respectively. The susceptible checks, Çağatay and Gökçe, had a disease severity and an incidence level of 2% and 6.67%, and 5.33% and 23.33%, respectively. In this method, Çağatay was resistant, while Gökçe was determined as moderately resistant. Among the resistance standards, Azkan was evaluated as the resistant (R) group with 1.93% disease severity or 10% disease incidence, and ICC3996 had no disease severity and disease incidence (**Table 4**). It was determined that the genotypes evaluated with the RT-PCR method in the study were 21% resistant (R), 35% moderately resistant (MR), and 44% susceptible.

**Table 4:** DNA amount (ng), disease severity %, disease incidence %, and disease classification in plants inoculated with the de-detached leaf inoculation method.

Genotypes	The amount of pathogen DNA	Disease severity %	Disease incidence %	Response	Genotypes	The amount of pathogen DNA	Disease severity %	Disease incidence %	Response
	(ng)					(ng)			
Tüb-01	3.23	23.33	73.33	S	Tüb-50	3.76	15.33	46.67	S
Tüb-02	20.55	56.19	90.48	S	Tüb-51	0.393	1.33	6.67	R
Tüb-03	4.66	16.67	70	S	Tüb-52	1.782	21.9	66.67	S
Tüb-04	4.86	24.67	76.67	S	Tüb-53	2.578	5.33	20	MR
Tüb-05	1.95	8	36.67	MR	Tüb-54	9.007	45.24	80	S
Tüb-06	0.28	8.67	36.67	MR	Tüb-55	1.145	2.67	13.33	MR
Tüb-07	1.26	8.67	40	MR	Tüb-56	0.215	2.67	13.33	MR
Tüb-08	4.47	10	33.33	MR	Tüb-57	0.509	6.67	23.33	MR
Tüb-09	0.28	11.33	30	MR	Tüb-58	17.7	28	63.33	S
Tüb-10	0.63	8.67	30	MR	Tüb-59	10.54	22	56.67	S
Tüb-11	0.41	2	10	R	Tüb-60	5.411	16.67	43.33	S
Tüb-12	0.37	2	10	R	Tüb-61	2.331	9.33	36.67	MR
Tüb-13	3.48	6	23.33	MR	Tüb-62	43.05	59.05	100	S
Tüb-14	0.08	8	40	MR	Tüb-63	12.94	26.67	76.19	S
Tüb-16	0.09	4	20	MR	Tüb-64	8.515	22.86	76.19	S
Tüb-18	3.88	62	90	S	Tüb-65	0.043	2	10	R
Tüb-19	0.12	4.67	23.33	MR	Tüb-66	50.6	96.19	100	S
Tüb-20	1.99	10.67	43.33	S	Tüb-67	65.4	72.38	90.48	S
Tüb-21	0.81	2	6.67	R	Tüb-68	8.964	37.14	76.92	S
Tüb-22	0.52	1.33	6.67	R	Tüb-69	0.1	0	0	R
Tüb-23	0.11	2.67	10	R	Tüb-70	0.37	2.67	13.33	MR
Tüb-25	0.15	2	10	R	Tüb-71	4.879	14	30	MR
Tüb-26	0.01	0.67	3.33	R	Tüb-72	80.96	72.81	96.67	S
Tüb-27	2.08	16.67	73.33	S	Tüb-74	11.32	100	100	S
Tüb-28	1.74	9.33	40	MR	Tüb-75	134.9	78.1	100	S
Tüb-29	1.22	5.33	23.33	MR	Tüb-76	9.479	21.9	61.9	S
Tüb-30	0.84	6	26.67	MR	Tüb-78	31.77	34.29	52.38	S
Tüb-31	5.53	12	43.33	S	Tüb-79	1.346	10	46.67	S
Tüb-33	1.28	14	56.67	S	Tüb-82	0.024	1.43	7.14	R
**Çağatay**	0.004	2	6.67	R	Tüb-84	12.43	44.05	100	S
**Gökçe**	1.15	5.33	23.33	MR	Tüb-86	3.479	39.17	91.67	S
Tüb-37	0.5	3.33	13.33	MR	Tüb-87	18.39	58.57	85.71	S
Tüb-38	6.71	6.67	20	MR	Tüb-93	11.32	37.14	100	S
Tüb-39	0.22	4.67	23.33	MR	Tüb-96	0.785	4	16.67	MR
Tüb-40	15.36	51.7	93.1	S	Tüb-97	16.86	24	40	MR
Tüb-41	0.22	14.67	70	S	Tüb-100	3.375	11.33	40	MR
Tüb-42	2.43	8.67	36.67	MR	Tüb-105	83.37	65.71	100	S
Tüb-43	0.16	2	10	R	Tüb-108	51.53	95.24	100	S
Tüb-44	0.57	0.67	3.33	R	Tüb-114	0.786	4	20	MR
Tüb-45	0.66	4	10	R	Tüb-119	2.245	29.52	100	S
Tüb-46	0.94	4.67	20	MR	Tüb-121	0.585	23.33	93.33	S
Tüb-47	0.01	0	0	R	Tüb-124	3.004	46	100	S
Tüb-48	2.15	14.67	60	S	**Azkan**	0.64	1.93	10	R
Tüb-49	4.55	10.67	30	MR	**ICC 3996**	0	0	0	R

Bold values are check (control) genotypes.

A positive relationship was determined between the amount of DNA (ng) of the pathogen in the leaf with the percent disease severity and disease incidence. The relationship between the amount of pathogen in the leaf and the disease severity (*r* ^2 =^ 0.53), as well as disease severity and disease incidence (*r* ^2 =^ 0.73) was significant.

However, the relationship between the pathogen amount in the leaf (ng) with the disease incidence % (*r* ^2 =^ 0.29) was not significant. The presence of the pathogen in the plant or an increase in the amount of DNA of the pathogen did not mean that the plant would be more severely diseased. While Tüb-14: 0.08 ng and Tüb-41: 0.22 had very little pathogenic DNA, the percentage of disease rate can be as high as 40% and 70%, respectively. In contrast, the amount of disease in Tüb-38 (6.71 ng) and Tüb-49 (4.55 ng), which have a high DNA content in the leaf, remained at a low level of disease incidence of 20% and 30%, respectively.

In the PCA for the four different methods applied in the study, 77.4% of the variation was covered by the first two components. For main component 1, MAS and AEC methods provide the highest positive contribution, whereas NFC contribution was found negative. In addition to the MAS and NFC methods, AEC has also made a positive contribution to main component 2. AEC in main component 3 and RT-PCR in main component 4 were the highest positive contributing method (**Table 5**).

**Table 5:** Variations and their components in the (PCA).

	Prin1	Prin2	Prin3	Prin4
MAS	0.79250	0.60575	−0.03842	0.05943
RT-PCR	−0.32159	−0.08415	−0.14658	0.93167
AEC	0.61147	−0.53588	0.57718	0.07617
NFC	−0.60506	0.64146	0.47131	0.01739

**Figure 4:**
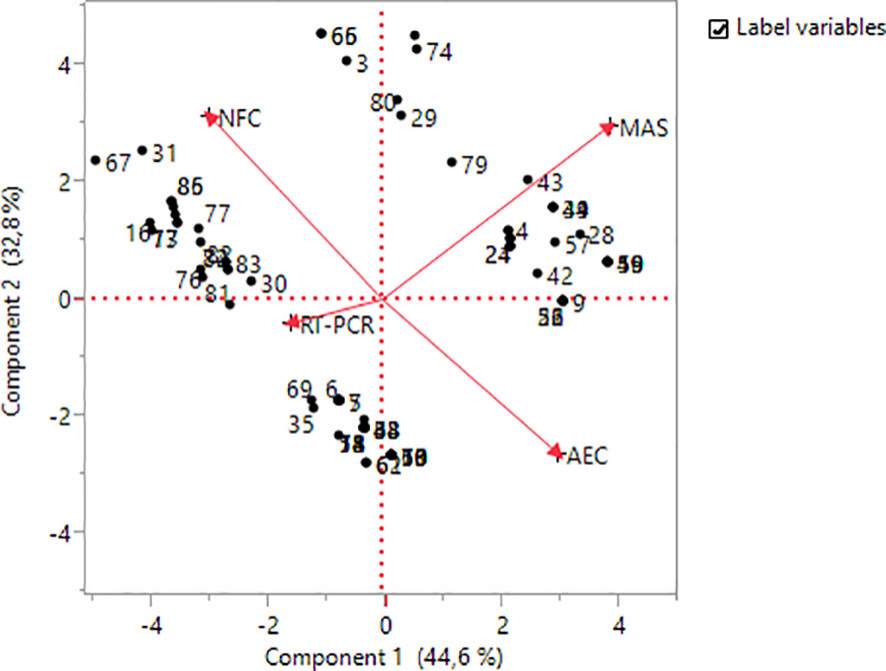
Biplot analysis for methods and genotypes.

In the Biplot analysis, it was seen that NFC and AEC, and MAS and RT-PCR, among the applied methods, had a negative relationship (**Figure 4**). When the distances of the methods from the origin were examined, it was seen that the highest variation was in NFC, MAS, and AEC, while the lowest variation was in RT-PCR. It was observed that genotypes numbered 31, 86, 77, and 67 were more related to the NFC method, genotypes numbered 43, 39, 4, and 24 were more related to the MAS method, and genotypes numbered 69, 30, 35, and 6 were more related to the RT-PCR method.

## 4 Discussion

The number of resistant genotypes was higher in the disease observation performed in NFC. In Haymana, the relative humidity (>60%) reached the most favorable values for the formation, development, and spread of the disease during the flowering and pod-filling period (June), when the plant is the most sensitive to AB. Pande et al. (2011a, b) also stated that the disease is more widespread in environments with high humidity (>60%). However, moisture is not a sufficient condition for the spread and occurrence of the disease. Temperature is also an important factor for the AB. In June, which is the flowering and pod-filling period in Haymana, the average temperature was 17.9˚C. This temperature value is below 20˚C, which is stated to be positively correlated with the occurrence of the disease (Trapero-Casas and Kaiser, 1992). In addition, temperature and humidity values can be considered appropriate under natural epidemics not noted in the early stage of growth, but during the later period, they increased resistance. Singh and Reddy (1993) also had similar observations and found susceptibility at later stages of plant development in the early part of crop growth, when relative humidity was high (>60%); cool temperatures (minimum < 5˚C and maximum < 15˚C) were found to limit blight epidemics. Among the AB disease screening methods examined, the most resistant material was found in NFC with 73%. The reason for this is that there are no suitable conditions (humidity, temperature, and precipitation) in the location when the plant is at the most sensitive stage.

In AEC, relative humidity and spring irrigation good for disease development was carried out after inoculation (Chen et al., 2005). In this study, Pathotype 1 was used for the inoculation. Different genotypes were defined as resistant, moderately resistant, and susceptible like many other studies (Singh and Reddy, 1993; Chen et al., 2004; Benzohra et al., 2013; Gayacharan et al., 2020).

Among the examined disease screening methods, the most susceptible number of genotypes was found in the AEC method with 84%. The results of the study are compatible with Gayacharan et al. (2020), who found that 10.6% of genotypes were susceptible and 87.4% were highly susceptible in their study in 1970.

The dilution or concentration of the inoculation source (spore suspension), the infection of different spores from the environment, the inoculated isolate, the type of pathotype, spores’ prevalence, aggressiveness, climatic conditions, time, and the number of applications of the inoculant influence the effectiveness of the method. Moreover, a positive correlation between field conditions and controlled environment screening technique for AB was reported by Pande et al. (2011a). In the AEC method, six lines were determined as resistant (Tüb 18, 19, 70, 71, 72, and 82). However, this resistance is not immune and is considered durable because it has a disease reading score between 1 and 3. Pastor et al. (2022) conducted a screening of 109 chickpea genotypes for *A. rabiei* under controlled conditions and determined that all genotypes were affected by the fungus.

In the MAS experiment, two QTLs (QTLAR1 and QTLAR2) having a relationship with AB disease were reported in a study including three SCAR markers (SCY19_336_, SCK13_603,_ and SCY17_590_) of QTLAR2, especially the SCK13_603_ marker, which is closely linked to the associated gene as cM, and it is recommended that this marker can be used primarily in susceptible/sensitive discrimination (Iruela et al., 2006). In the study, both markers showed similar efficacy, and the SCY17_590_ marker determined the genotype with a higher number of resistance alleles differently. When the markers SCY19_336_, SCK13_603_, and SCY17_590_ scanned in agarose gel electrophoresis were compared among themselves, it was seen that all of these markers showed the presence of the resistance allele. Resistance could not be determined in at least one of the three SCAR markers in materials with moderate resistance. In the study with three markers, genotypes showing resistance allele in all markers reached 61% of the total genotype. This amount is close to durability (73%) in natural conditions. The number of susceptible genotypes in MAS was lower than that in AEC and suspicion regarding the efficiency of the marker increased. However, Ali et al. (2008) used three SCR (SCY603, 590, and SCADA SCY19) markers for the screening of 21 local chickpea genotypes and noted that one STMS marker (TA 146) and three SCAR markers (SCAE19_336_, SCK13_603_, and SCY17_590_) covering the distance of 0.5 cM on this linkage group were linked with resistance in genotypes. The genome walking method used in a study was useful to sequence flanking regions of the marker SCK13_603_ tightly linked to QTLAR2 for AB resistance (Iruela et al., 2009). Some studies are compatible with our MAS findings but contradict our AEC findings. One allele-specific marker (CaETR) and one codominant SCAR17_590_ marker were reported to have contributed efficiently to the selection of new chickpea varieties with better combinations of alleles to ensure durable resistance to the AB (Bouhadida et al., 2013).

Two of the four SCARs showed significant alignment with genes or proteins related to disease resistance in other species and one of them (SCK13_603_) was cited in the highly saturated region linked (Iruela et al., 2006). It is determined that it is resistant to ICC3996 Pathotypes 1 and 2 (Chen et al., 2004) and is compatible with the findings of this study, while susceptible checks (Gökçe and Çağatay) have resistance allele in three SCAR markers, and they are identified as susceptible in AEF and NFC. A study with 23 Tunisian chickpea genotypes found that the V10 line showed a resistance allele in CaETR and was heterozygous for SCAR17_590_; it is moderately resistant under natural conditions and controlled conditions (Bayraktar et al., 2016). The results are comparable to these findings. None of the various AB resistance QTLs have been reported to be used in MAS.

Several screening techniques under field and controlled environments have been reported for AB (Pande et al., 2005). Resistant cultivars are difficult to obtain due to the continuous evolution of the fungus and the appearance of new pathotypes that overcome the resistance of existing cultivars. In addition, disease resistance is considered a quantitative trait and numerous QTLs have been identified on the chickpea genetic map (Millàn et al., 2010). Breeders are attempting to combine genes in new cultivars to improve the level and durability of resistance, but this process is further complicated when different QTLs or genes control the same phenotype. SCAR markers have some advantages such as being highly reproducible, quick and simple, and locus-specific; however, they also have disadvantages such as the need for gene sequence to design markers and sometimes radioactive isotopes are required (Dar et al., 2019).

Traditional methods of isolation and identification of *A. rabiei* are time-consuming. Polymerase chain reaction (PCR) techniques offer advantages over traditional plant disease diagnosis because organisms do not need to be cultured before detection by PCR. RT-PCR has been referred to as a rapid, sensitive, and specific method for pathogen detection and the evaluation of host resistance, epidemiological studies, and disease management (Schaad and Frederick, 2002; Schena et al., 2004).

Although there is a relationship between the amount of DNA of the pathogen in the leaf and the rate of disease, it has been observed that this is not very important because genotypes have both active and passive defense responses to stop initial pathogenic attacks and to prevent successful invasion and spread to neighboring cells (Coram and Pang, 2006). Passive defense mechanisms include preformed structural and chemical barriers such as glandular trichomes, which secrete antifungal isoflavones (Armstrong-Cho and Gossen, 2005). Active defense systems in plants may employ R genes to recognize pathogen-specific effectors encoded by the Avr genes (McDonald and Linde, 2002), leading to effector-triggered immunity (ETI) and possible programmed cell death (PCD) via a hypersensitive response (HR) (Jones and Dangl, 2006). RT-PCR is compared to another screening method, AEC; while the number of resistance materials in the RT-PCR method is 18, this amount is only 8 in AEC. In addition, only three materials (Azkan, Tüb-18, and ICC3996) were found resistant in the RT-PCR technique in the AEC screening method.

The PCR-based method developed can simplify both plant disease diagnosis and pathogen monitoring in an early phase, as well as aid in effective management practices that avoid disease advancement and minimize losses (Valetti et al., 2021). RT-PCR has many advantages over conventional PCR: (1) it does not require the use of post-PCR processing, (2) it avoids the risk of cross-contamination, (3) it reduces assay labor and material costs, and (4) it increases sensitivity and specificity and allows accurate quantification of the target pathogen (Kumar et al., 2016).

On the other hand, the RT-PCR technique has some disadvantages such as contamination of the plant tissues by spores of the pathogen. This is because genotypes have both active and passive defense responses to stop initial pathogenic attacks, prevent successful invasion, and spread to neighboring cells (Coram and Pang, 2006). This could have occurred during the sampling of tissues for the analysis, or naturally by spores transported on the surface of the trunk (Chandelier et al., 2018). Furthermore, conventional lab-based PCR technology requires expensive laboratory equipment and skilled personnel, which is a major disadvantage in adopting this technology as a detection method for on-site purposes (De Shields et al., 2018).

In this study, four different screening methods, namely, NFC, AEC, MAS, and RT-PCR, are used in the selection of AB disease resistance for chickpea genotypes. No genotypes identified as common S and MR were found in all four different screening methods used in the study. However, Azkan, ICC3996, Tüb-19, and Tüb-82 were determined as resistant within all methods for Pathotype 1.

Among the examined screening methods, significant differences occurred in the level of resistance and number of genotypes in expression for AB disease. It was determined that the most effective method among the screening methods was AEC. Resistance to AB of the genotype is one of the most important selection criteria for the chickpea breeding strategy. The method of material selection for the disease should be effective, accurate, fast, and economical.

The occurrence of the disease in the NFC method depends on the environmental conditions, and there is an uncertainty of the inoculation source. In the AEC method, it is partially dependent on environmental conditions, and there may be isolates other than the given inoculant. In the MAS method, there is a lack of an effective marker for the environment and genotypes cannot be defined precisely with markers for the disease. These facts make it useful for the early detection of infected tissues in the RT-PCR method. Considering all these unfavorable conditions, it was concluded that using fully controlled environmental conditions and artificial inoculation is the most effective method for screening chickpea genotypes in the AB disease evaluation.

## Data availability statement

The original contributions presented in the study are included in the article/supplementary files, further inquiries can be directed to the corresponding author/s.

## Author contributions

AA: Writing – original draft.
